# Mirroring minds: assessing the relative stability of self-appraisal and reflected appraisal in daily life

**DOI:** 10.3389/fpsyg.2025.1576353

**Published:** 2025-04-25

**Authors:** Isabel Dorothee Gütges, Haoran Xi, Siegfried Gauggel, Saskia Doreen Forster

**Affiliations:** Institute of Medical Psychology and Medical Sociology, University Hospital of the RWTH Aachen, Aachen, Germany

**Keywords:** self-appraisal, reflected appraisal, stability, daily life, ecological momentary assessment

## Abstract

**Introduction:**

Cultivating a stable self-concept is vital for mental and social well-being. Instability in the processing of self-related information, particularly concerning self-views, have been associated with various mental disorders. Central to the stability of self-perception are two key constructs: self-appraisal and reflected appraisal. Self-appraisal refers to individuals’ personal evaluations of their worth, while reflected appraisal encompasses beliefs about how one is perceived by others. Although previous laboratory studies have examined the formation and impact of self-appraisal and reflected appraisal on self-concept, fluctuations in reflected appraisal relative to self-appraisal in daily life remain largely unexplored. This study aimed to address this gap by examining the variability of both appraisal types and their association with mood changes in everyday contexts.

**Methods:**

Utilizing ecological momentary assessment, student participants reported their self-appraisal, reflected appraisal, and mood eight times daily over a ten-day period.

**Results:**

The analysis revealed that self-appraisal exhibited between-subject (ICC = 0.70) and within-subject (ICC = 0.30) variability. Also, reflected appraisal demonstrated between-subject variability (ICC = 0.72) and within-subject variability (ICC = 0.28). Notably, the results indicated that self-appraisal fluctuated more significantly than reflected appraisal (*t* = 2.58, df = 98, *p* = 0.01). Furthermore, a moderate correlation was observed between self-appraisal variability and mood variability (*r* = 0.45, *p* < 0.01), whereas the correlation for reflected appraisal variability was weaker (*r* = 0.35, *p* < 0.01).

**Discussion:**

These findings underscore the distinct fluctuation patterns of self-appraisal and reflected appraisal in daily life, suggesting that reflected appraisal serves as a stabilizing anchor for self-concept consistency. This study provides a crucial foundation for future research on normative stability within the self-concept framework.

## 1 Introduction

Individuals actively seek to construct and sustain their self-concepts in relation to both themselves and others, making the cultivation and maintenance of a stable self-concept essential for mental and social well-being (Church et al., 2014; Gecas et al., 1995). The self-concept refers to the organized set of beliefs and perceptions individuals hold about themselves, shaping their behavior, experiences, and interactions (Mann et al., 2004; Vallacher et al., 2002). The self-concept encompasses mental representations of oneself and is critical for self-confidence, efficacy perceptions (Kleitman and Stankov, 2007; Vega et al., 2020) and self-regulation as it provides individuals with a stable framework to evaluate and adjust their thoughts, emotions, and behaviors in alignment with their goals. It recruits higher-order self-reflective cognitive processes that enable individuals to evaluate their patterns of thinking, feeling, and behaving, as well as social cognitions of how others perceive those patterns (Carlson et al., 2010; Flavell, 1979). Alterations in processing self-related information, especially unstable self-views, have been linked to various mental disorders such as Post-traumatic stress disorder (Kashdan et al., 2006) Depression (Oosterwegel et al., 2001; Strauman, 2014), Schizophrenia (Pauly et al., 2014), and Borderline-personality disorder (Santangelo et al., 2017a; Winter et al., 2017).Unstable self-views undermine an individual’s ability to maintain consistent self-regulation, emotional stability, and a coherent sense of identity, emphasizing the importance of understanding the factors that contribute to its stability.

Understanding the stability of self-perception is crucial as it influences how people construct their self-concept and manage interpersonal interactions. Stable self-perception fosters self-consistency (Epstein, 1979) and contributes to the experience of unity, independence, predictability, and control (Barbin and Ninot, 2008). Central to understanding the stability of self-perception are two related constructs: self-appraisal and reflected appraisal. Self-appraisal refers to an individual’s own evaluations of their characteristics, abilities, and worth, derived from self-reflection on internal criteria (e.g., values, self-knowledge) and personal experiences (Culatta, 2018; Rosenberg, 1989). In contrast, reflected appraisal pertains to how individuals believe that they are perceived by others. Reflected appraisal is formed by one’s self-perception and interpretation of social feedback and social behaviors (Jaret et al., 2005; Shrauger and Schoeneman, 1979; Wallace and Tice, 2012). While these constructs are integral to self-concept, their stability in daily life remains insufficiently explored.

Whereas global evaluations of self-views are generally stable over time, individuals’ self-views exhibit considerable variability due to immediate contexts, emotions, and external feedback (Brown and Mankowski, 1993; Kernis and Goldman, 2013; Oosterwegel et al., 2001; Trzesniewski et al., 2003). This variability underscores the sensitivity of state self-views to momentary influences. While empirical research has shown significant fluctuations in self-appraisal, the stability of reflected appraisal in everyday life remains largely unexplored. Laboratory studies have demonstrated that individuals often view themselves more positively from the perspective of close others compared to their own perspective (Forster et al., 2019). However, the degree of stability or instability of reflected appraisal in naturalistic settings has not been thoroughly examined. Understanding this stability is essential for gaining insights into normative patterns of self-perception and its implications for mental well-being.

Concerning the stability of self-appraisal and reflected appraisal existing research suggests that individuals’ perceptions of the self and how they believe others perceive them, follow distinct patterns of stability shaped by their respective sources of information and formation processes (Wallace and Tice, 2012). Although both self-appraisal and reflected appraisal are constructed by the same individual, they represent different perspectives on the self – the former from an internal viewpoint and the latter from a perceived external viewpoint. Empirical studies support these differences: for example, Oosterwegel et al. (2001), using a daily diary approach, demonstrated that individuals’ self-appraisal fluctuates in accordance with changes in mood, highlighting the influence of transient internal states. In contrast, research by Carlson et al. (2011), using peer-report and self-other agreement methods rather than a daily diary approach, showed that reflected appraisal is more stable and grounded in consistent social feedback. These findings underscore that while self-appraisal may be more dynamic, reflected appraisal tends to offer a more stable perspective on the self.

To understand these differences more fully, it is essential to consider the mechanisms through which self-appraisal and reflected appraisal are formed. When individuals appraise themselves and their behavior, they have access to internal inputs (inputs that others lack access to) that are more likely to be affected by momentary changes such as those brought about by changes in mood (Brown and Mankowski, 1993; Geukes et al., 2017; Nezlek and Plesko, 2001). This can be understood through the lens of the Emotion-as-Information Theory (Schwarz, 2011), which suggests that individuals use their current emotional states as information to guide their judgments and decisions. As a result, self-appraisal can vary more dramatically than global self-perceptions as they interpret their emotions to evaluate their self-worth. Studies that have assessed peoples’ self-appraisal and mood in daily life confirm this finding by indicating that when in a positive mood, individuals are more likely to interpret their self-worth in a favorable light, whereas a negative mood can lead to more critical self-evaluations (Forster et al., 2022; Oosterwegel et al., 2001). This highlights the dynamic nature of self-appraisal and their susceptibility to fluctuations in emotional states, illustrating how momentary changes in mood can influence one’s evaluation of their self-worth.

While self-appraisal is shaped by internal emotional states and thus prone to fluctuation, reflected appraisal may similarly be influenced by changes in mood. However, since it is formed by external social feedback and psychological distance, it potentially offers a more stable and objective evaluation of the self. Research by Carlson et al. (2011) indicates that individuals possess interpersonal perceptions skills that allow them to gauge how they are seen by others, enhancing their understanding of the self. Reflected appraisal involves considering perceptions from various social sources including specific individuals such as friends, larger groups like family, and even broader societal norms (Natanson, 2012, p. 72). Adopting a third-person perspective serves to create psychological distance, which encourages abstract thinking compared to a first-person viewpoint (Libby et al., 2005). Observing oneself from this detached viewpoint has been linked to greater objectivity in self-evaluation (Zhou et al., 2017), to obtain lower emotional experience (Berntsen and Rubin, 2006), and decreased egocentric bias (Zhou et al., 2013). These factors suggest that reflected appraisal is subject to fluctuation. However, by incorporating psychological distance, it may be less influenced by transient emotional states, thereby offering a more stable assessment of the self compared to self-appraisal.

Building on the idea that reflected appraisal is shaped by external inputs and psychological distance, it is also influenced by consistent social feedback over time, which conceivably further reinforces their stability. Reflected appraisal are shaped by relatively consistent social feedback and generalized impressions accumulated over time (Felson, 1985; Kinch, 1963), which serve as a fundamental element of self-concept development. Social perceptions tend to aggregate and stabilize through repeated interactions, providing individuals with a reliable framework for self-understanding (McCall and Simmons, 1978). Theories of social comparison and validation suggest that individuals actively seek consistency and coherence in how they perceive themselves relative to others (Festinger et al., 1954; Swann, 2012), fostering stable reflected appraisal as they align their self-views with perceived social consensus. This stability ensures that reflected appraisal provide a more consistent reference point derived from social interactions.

*Hypothesis 1*: Reflected appraisal show less variability than self-appraisal in daily life.

This hypothesis posits that reflected appraisal exhibits greater stability than self-appraisal, grounded in its dependence on consistent social feedback and accumulated impressions from external sources over time. In contrast to self-appraisal, which is influenced by momentary mood fluctuations, reflected appraisal benefit from psychological distance and is shaped by repeated interactions and prevailing social norms. Despite its reliance on stable external sources, reflected appraisal may still exhibit some fluctuation due to individuals’ interpretations of others’ perceptions, which can be influenced by internal cognitive and emotional processes. Nevertheless, these factors suggest that reflected appraisal offers a more stable and objective reference for self-evaluation, as they are less susceptible to the day-to-day emotional variability inherent in self-appraisal.

Conversely, self-appraisal contributes to a flexible and adaptable self-concept, responsive to immediate experiences and contexts. Thus, self-appraisal is inherently more variable as it fluctuates with moods and internal changes, reflecting the dynamic nature of an individual’s self-evaluation.

*Hypothesis 2*: Both instability in reflected appraisal and self-appraisal are positively associated with instability in mood; however, the association between instability in mood and the instability in self-appraisal is stronger than the association between the instability in mood and the instability in reflected appraisal.

This hypothesis acknowledges that while both types of appraisal instability can be linked to mood fluctuations, the more pronounced effect on self-appraisal highlights their susceptibility to daily emotional states. Despite the theoretical foundations suggesting the greater stability of reflected appraisal, empirical research on this aspect remains limited. Previous studies have extensively examined the formation and impact of self-appraisal and reflected appraisal on self-concept (Asencio, 2013; Asencio and Burke, 2011; Granberg, 2011; Leary et al., 2000; Marcussen and Asencio, 2016; Shrauger and Schoeneman, 1979; Tice and Wallace, 2003), but the relative stability of these constructs in everyday contexts has not been rigorously investigated. Understanding the relative stability of self-appraisal and reflected appraisal can shed light on normative patterns of self-perception.

## 2 Methods

### 2.1 Participants

Participants were recruited between May and June 2023 via flyers on the University Campus. Additionally, online flyers were sent through e-mail to participants that took part in previous studies of the department and subscribed to receive study invitations. To ensure adequate power for detecting at least a medium effect size (d = 0.5), *a priori* power calculations were conducted using the *PowerCurves* tool (Kleiman, 2021). Based on a 10-day data collection period with 8 measurement points per day, the calculations suggested that a minimum of 100 participants was necessary to achieve approximately 80% power for detecting a medium effect size, as indicated by the power curve for d = 0.5 (Maas and Hox, 2005; Bolger et al., 2011). Participants were included if they were between the age of 18 and 50 years, German-speaking and owned a smartphone with android operating system to meet the compatibility requirements of the electronic Ecological Momentary Assessment application *MovisensXS* (Movisens GmbH, Karlsruhe, Germany) used in this study. Participants who self-reported suffering from a current neurological or mental disorder were excluded. 100 Participants agreed to take part and gave written consent. Only one of them was not included in the data analyses, due to an early drop out. Of the 99 participants, 53 were males (53.5%) between the age of 18 and 44 (mean age = 25.1 years, SD = 4.24) and 45 were females (45.5%) between the age of 18 and 44 (mean age = 24.8 years, SD = 5.11). One 19-year old participant identified as divers (1%). Out of the total participant pool, 84 individuals were identified as students (84.9%) among whom 24 (24.3%) were concurrently engaged in employment. Additionally, 15 participants were employed (15.2%) without concurrent enrollment in an academic program. See Table 1 for details.

**Table 1:** Descriptions of the sample with Means (M) Standard Deviations (SD), Frequencies (n), and Percentages (%).

Variable	Category	Participants (*n* = 99)
Age, *M* (SD)	Years	24.88 (4.7)
Gender, *n* (%)	Female	45 (45.5)
RSES *M* (SD)	Baseline measure	3.1 (0.49)
Self-reports *M* (SD)	Completed	71.72 (6.89)
Compliance rate (%)		89.65 (8.62)
Response time *M* (SD)	Seconds	61.1 (69)
Response delay *M* (SD)	Seconds	58 (151.8)

RSES, Rosenberg Self-esteem Scale.

### 2.2 Procedure

The recruitment started with a short e-mail contact to check if the inclusion criteria (see above) were met. Potential participants were separately invited to a briefing on site. During the briefing, each participant received verbal and written descriptions of the purpose and procedure of the study, and all participants’ written informed consent was obtained. The study protocol (Protocol EK 22–325) was approved by the local ethics committee in accordance with the Declaration of Helsinki. Thereafter, they were instructed to download the application *MovisensXS* version 1.5.23 (Movisens GmbH, Karlsruhe, Germany) on their personal smartphones and asked to fill out the “Rosenberg Self-esteem Scale” (Rosenberg, 1965) within the app. Then, the participants were familiarized with the EMA-method and given instructions on how to answer the daily study questions on their smartphone. Data was collected in the course of 10 consecutive days and started on the following day, after the individual instructions had been given. Participants were prompted to respond 8 times a day between 8 a.m. and 10 p.m., at random moments (with a minimum break of 30 min between following beeps) to questions about spontaneous thoughts, affective states, self-appraisal, reflected appraisal, and social context. The prompts were received through vibrations and sound via the smartphone. Immediately after a prompt was given, participants had the option to postpone responding to the questions to 5, 10 or 15 min in case answering was not practicable in the moment of the prompt. After 20 min the prompt was closed. Participants were prompted a maximum of five times for each assessment. They entered their responses into the MovisensXS application on their smartphones. The responses were not stored locally on the devices but transmitted via end-to-end encrypted connections to a secure central database for subsequent analyses. Throughout the study period, responses were monitored for completeness and technical issues. In the event of technical problems, troubleshooting support were provided by the researcher via a chat in the app. Participants did not receive reminders for missed prompts, but they were given regular feedback on the number of questionnaires they had completed. Missed prompts could not be repeated. Participants were financially compensated for their participation in the study, depending on the number of questionnaires that they completed. For a completion rate of at least 50%, they received 35€, for 75% 50€ and for 90% 75€.

### 2.3 Measures

All measurements were in German language and assessed via the software *movisensXS*, App version 1.5.23 (Movisens GmbH, Karlsruhe, Germany). The questions, as they were asked in German, the English translation, as well as further baseline and state measurements that were part of a broader research agenda are available in the Supplementary material.

#### 2.3.1 Ecological momentary assessment

The EMA included 15 questions in total, that were divided into four subsets. The chronological order of these following subsets remained the same throughout each prompt with the exception of the scales of self-appraisal and reflected appraisal. The presentation of these two scales were changing randomly at each beep to avoid sequence effects.

##### 2.3.1.1 Mood

Based on the guidelines from Wilhelm and Schoebi (2007), the construct of mood is anchored in three fundamental dimensions: energetic arousal, calmness, and valence. For the valence dimension, the Ecological Momentary Assessment (EMA) version from Simons et al. (2020) was used, which measures affect using seven items. These include three items for positive affect (content, happy, cheerful) and four items for negative affect (insecure, down, guilty, afraid). An example of a positive affect item is, “In this moment I feel content,” while an example of a negative affect item is, “In this moment I feel down.” Each item was rated on a seven-point Likert scale, ranging from 0 (Not at all) to 6 (Very). For the dimensions of energetic arousal and calmness, two bipolar items were rated on a similar seven-point Likert scale: from 0 (without energy) to 6 (full of energy) for energetic arousal, and from 0 (very tense) to 6 (very relaxed) for calmness. To summarize these dimensions, the mean response for each participant at each prompt was calculated, resulting in a composite score for each of the three mood facets. These were then combined into a single composite mood score, as they reflect complementary aspects of the broader mood construct (Wilhelm and Schoebi, 2007).

##### 2.3.1.2 Self-appraisal and reflected appraisal

To assess participants current self- and reflected appraisal, four items of the “Rosenberg Self-esteem scale” (Von Collani and Herzberg, 2003) were used. The choice of items was based on previous studies that adapted the RSES scale (Santangelo et al., 2017a; Santangelo et al., 2020) to fit the EMA-protocol. Participants were presented with the question “How do you see yourself at this moment?” followed by four items (1) “I think, that I am a failure.” [“Ich halte mich für einen Versager.”], (2) “I am satisfied with myself.” [“Ich bin mit mir selbst zufrieden.”], (3) “I think I am no good at all.” [“Ich denke, dass ich gar nichts tauge.”], and (4) “I consider myself a person of worth.” [“Ich halte mich für einen wertvollen Menschen.”]. To measure reflected appraisal this four-item scale was adapted to assess the self from others’ perspective. Participants were presented with the question “How would others appraise you at this moment?,” followed by the four items (1) “Other People think that I am failure” [“Andere Menschen halten mich für einen Versager.”], (2) “Others are satisfied with me.” [“Andere Menschen sind mit mir zufrieden.”], (3) “Others think that I am no good at all.” [“Andere Menschen denken, dass ich gar nichts tauge.”], “Others think I am a valuable person.” [“Andere Menschen halten mich für einen wertvollen Menschen.”]. All items were answered on a 10-point Likert-scale ranging from 0 = “does not apply at all” to 9 = “applies fully.” The 10-point scale was selected as it allows for a more detailed measure of self-appraisal and reflected appraisal, capturing subtle variations and context-specific aspects of self-evaluation, as demonstrated in previous studies using the RSES in Ecological Momentary Assessment (EMA) (Santangelo et al., 2017a; Santangelo et al., 2020). The reliability of self-appraisal was good with α = 0.81 and of reflected appraisal α = 0.8. Additionally, the convergent validity of the self-appraisal measure was strong, as indicated by a Spearman correlation of *r* = 0.77 with the trait measure, the Rosenberg Self-Esteem Scale (RSES). Although these measures are not identical, the state item demonstrates convergence with the trait measure, suggesting that it effectively captures state-specific aspects that differ from the trait.

### 2.4 Data analysis

All analyzes were performed with R software (R Core Team, 2020) and the RStudio (RRID:SCR_000432) and R Project for Statistical Computing (RRID:SCR_001905) integrated development environment [version 4.3.2, RStudio Team, (2024)] using the packages *psych* (Revelle, 2018), *lme4* (Bates et al., 2014), and *Hmisc* (Harrell and Dupont, 2020).

#### 2.4.1 Data preprocessing

Composite scores for self-appraisal, reflected appraisal, valence, tense arousal and energetic arousal were computed by calculating the mean values of the respective items for each administration of the scale. The items negative affect, self-appraisal, and reflected appraisal with a negative valence of these scales were reverse coded. Thus, higher scores corresponded to a positive state for all scales (e.g., positive self-appraisal, good mood, great calmness, or low tense arousal).

#### 2.4.2 Fluctuations

To determine the source of variability the Intraclass Correlation Coefficient (ICC) of self-appraisal, reflected appraisal, and mood was determined separately to assess how much variance in the measurement is attributable to the differences between subjects as opposed to the variance due to measurement error (within subjects).

To attain response fluctuation, each participant’s root mean square of successive differences [rMSSD (Jahng et al., 2008)] for each day of each Participant was calculated separately for self-appraisal, reflected appraisal, and mood. This instability index allows for examining group differences while taking into account the temporal dependency of the unstable processes (Jahng et al., 2008). This was done by calculating the differences between consecutive assessments (excluding nights), squaring these differences, taking the mean of the squared differences and finally taking the square root of that mean to ensure that the rMSSD values were on the same scale as the original data. Therefore, larger differences between consecutive assessments are emphasized more compared to smaller differences. Differences between the rMSSD of self-appraisal and the rMSSD of reflected appraisal were analyzed with a paired t-test. In addition, this difference was confirmed with a multilevel model, where the fixed effects captured the general relationship between the predictor (appraisal type) and the dependent variable (rMSSD), while the random effects accounted for the individual variations in this relationship. For interpreting the results, the focus was solely on the fixed effects, as the random effects were included merely to account for heterogeneity in the data and were not central to the research question. Spearman correlation analyses were conducted to examine the associations between the rMSSD of self-appraisal, mood, and reflected appraisal.

To investigate acute changes in self-appraisal, reflected appraisal, and mood, a logistic regression model with a logit link function was employed, following the approach described by (Jahng et al., 2008; Santangelo et al., 2017b). This model quantified the “probability of acute change” [PAC, (Jahng et al., 2008)] for each variable and facilitated comparisons of their effects. The 90th percentile of each distribution was used as a cut point for acute increases and the 10th percentile of each distribution was used as a cut point for acute decreases. Fixed effects included self-appraisal, reflected appraisal, and mood as binary predictors to assess their impact on PAC. Random intercepts for participants were included to account for variability in baseline PAC across individuals. This multi-level model considered the different levels of measurement and provided insights into how self-appraisal, reflected appraisal, and mood influence the likelihood of experiencing acute changes.

## 3 Results

### 3.1 Descriptive statistics

Participants answered on average 71.7 (*SD* = 6.9) of the 80 prompts (Table 1). The number of prompts varied among the participants, ranging from 37 to 80 prompts. The compliance rate of 89.6% did not decline over the assessment period [*b* = 0.00, *SE* = 0.00, *p* = 0.3 (*p >* 0.05)].

### 3.2 Analyses of variability

Regarding the source of variability in participants’ self-appraisal, 69.9% of the variance in participants’ responses [95%CI (1.13, 1.51)] was due to differences between participants and 30% [95 CI (0.85, 0.87)] was due to differences within participants. For reflected appraisal, 71.7% of the variance [95 CI (1.12, 1.49)] was explained by differences between participants and 28.3% [95 CI (0.80, 0.82)] was due to differences within participants. With regard to mood, 24% of the variance [95 CI (0.55, 0.75)] was due to differences between participants and 76% of the variance [95 CI (1.13, 1.16)] was due to differences within participants.

### 3.3 Difference between self-appraisal instability and reflected appraisal variability

**Figure 1:**
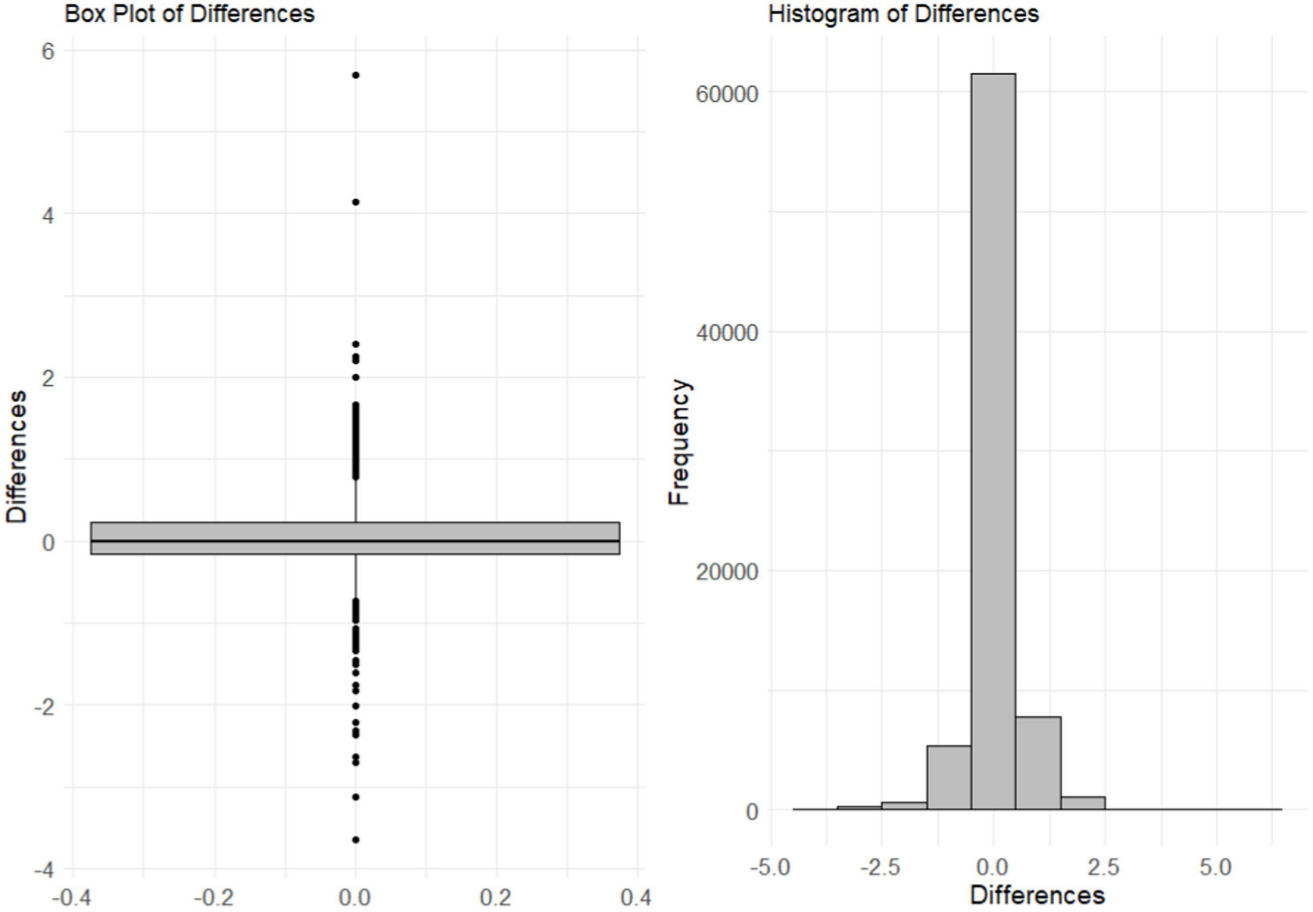
Differences between rMSSD of self-appraisal and reflected-appraisal. This figure combines a histogram and a boxplot of the differences between the rMSSD of self- and reflected-appraisal. The mean difference is significant *p* = 0.01.

The paired t-test revealed a significant difference between the rMSSDs of self-appraisal and reflected appraisal (*t* = 2.58, *df* = 98, *p* = 0.01, *MD* = 0.05) (see Figure 1). The multilevel model supports this finding (𝛽 = − 0.06, *SE* = 0.00, *p* = 0.00). In addition, rMSSD of self-appraisal and reflected appraisal result in a significant, strong and positive correlation (*r* = 0.69, *p* < 0.01).

### 3.4 Associations of instabilities of self- and reflected appraisal and mood

The rMSSD of self-appraisal correlated moderately with the rMSSD scores of mood (*r* = 0.45, *p* < 0.01). This indicates that participants who showed high variability in self-appraisal, also showed high variability in mood. In addition, a significant, weak and positive correlation was found between the rMSSD of reflected appraisal and mood (*r* = 0.35, *p* < 0.01). Thus, participants that showed higher variability in their reflected appraisal also showed higher variability in their mood. Nevertheless, the correlation between the rMSSD of self-appraisal and the rMSSD of mood was significantly stronger than the correlation between the rMSSD of reflected appraisal and rMSSD of mood (*z* = 2.44. *p* = 0.02).

**Figure 2:**
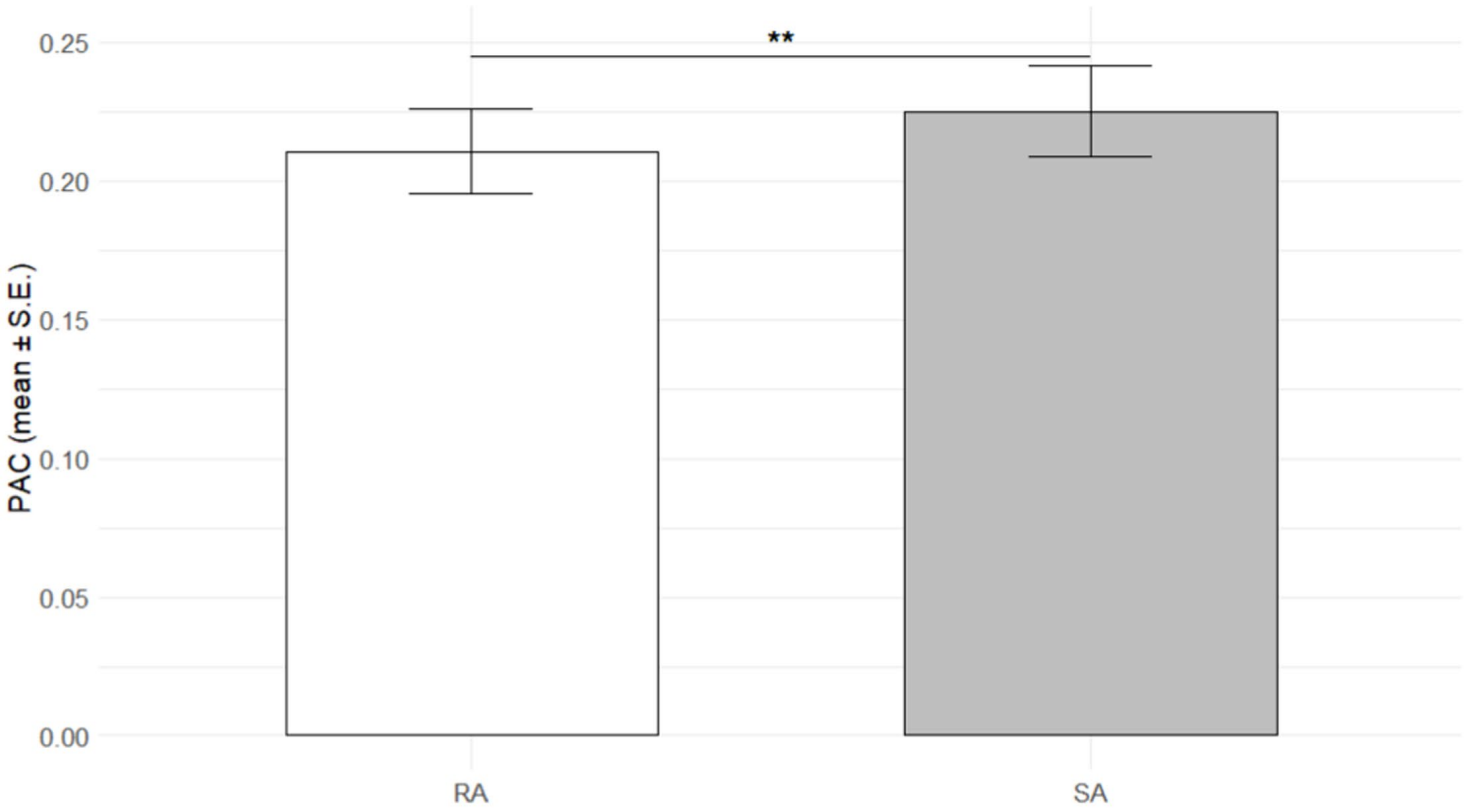
Barplot of the propability of acute change (PAC) per appraisal type. PAC, Probability of acute change, i.e., the number of acute changes divided by the total number of changes, separate for self-appraisal (SA) and reflected appraisal (RA). ** *p* < 0.01. Significance levels of group differences respresent the results of the multilevel models reported in the text.

The multilevel PAC analysis is in line with the rMSSD finding since the fixed effects of the model indicated that the probability of acute change was significantly lower for reflected appraisal compared to self-appraisal [PAC: 𝛽= − 0.10, *SE* = 0.04, *z* (15503) = −2.51, *p* = 0.01]. This result suggests that individuals were less likely to experience acute changes during reflected appraisal compared to self-appraisal. The odds ratio for this effect was 0.90, indicating a reduced likelihood of acute change during reflected appraisal by approximately 10% [95% CI (−0.186, −0.023)] compared to self-appraisal. The result is consistent with the hypothesis that self-appraisal might be more sensitive to sudden shifts compared to reflected appraisal. Figure 2 depicts the mean PAC of all participants for reflected appraisal and self-appraisal.

Further, multilevel PAC analyses indicated that probability of acute change in self-appraisal was not significantly different from the probability of acute change in mood [PAC: 𝛽 = − 0.02, *SE* = 0.04, *z* (15503) = −0.48, *p* = 0.629]. This result suggests that there is no significant difference in the likelihood of experiencing an acute change between self-appraisal and mood. The odds ratio for this effect is 0.98, indicating a 2% decrease in the likelihood of experiencing an acute change during self-appraisal compared to mood assessments, but this result is not statistically significant [95% CI (−0.08, 0.06)]. However, another PAC model indicated that the probability of an acute change in mood was marginally higher compared to reflected appraisal [PAC: 𝛽 = 0.08, *SE* = 0.04, *z* (15503) = 1.95, *p* = 0.05]. This result suggests that individuals were slightly more likely to experience acute changes during mood assessments compared to reflected appraisal. The odds ratio for this effect was 1.08, indicating an 8% increase in the likelihood of experiencing an acute change during mood assessments compared to reflected appraisal, although this result is only marginally significant [95% CI (−0.01, 0.17)].

## 4 Discussion

The present study is the first that longitudinally assessed reflected appraisal across several days employing an EMA approach and providing insight into the temporal stability of reflected appraisal relative to self-appraisal. The findings indicated that similar to self-appraisal, reflected appraisal varied within and between participants. For both appraisal types, about 30% of variance was explained by within-person variability. This is a moderate proportion illustrating the degree of day-to-day changes. Although, in intensive longitudinal studies involving self-reports of psychological constructs it is usual to have within variability in the 0.6–0.8 range (Bolger and Laurenceau, 2013). However, the ICC of self-appraisal was similar to other studies assessing self-esteem with similar items in daily life (Crowe et al., 2019). Moreover, approximately 91.5% of the self-appraisal rMSSD values and 88.8% of the reflected appraisal rMSSD values indicated some degree of temporal instability (rMSSD > 0) during the observational period.

Further, the findings of the in-depth analysis of timely patterns confirm the expectations that reflected appraisal exhibit less variability than self-appraisal and demonstrate, that how people evaluate themselves changes more dynamically over time compared to their beliefs of how others evaluate them. Although reflected appraisal and self-appraisal are correlated, their differences in variability highlight that they are not entirely interchangeable. The PAC analysis emphasized this difference by showing that the estimated odds of acute change in appraisal indicate a 10% higher risk of an acute change in self-appraisal relative to reflected appraisal. These findings support the idea that self-appraisal is more prone to radical change. This was the case for acute changes of rapid increases as well as rapid decreases. Thus, relative to self-appraisal reflected appraisal is characterized by slower repairs of self-evaluation but also by slower worsening. These findings align with research on perspective-taking, where reflecting on how others perceive oneself involves adopting a broader and more detached view of the self, one that is less influenced by internal emotional fluctuations (Zhou et al., 2017; Berntsen and Rubin, 2006). From a developmental standpoint, reflected appraisal are shaped by relatively consistent social feedback and the generalized impressions that accumulate over time (Felson, 1985; Kinch, 1963). Therefore, they serve as a fundamental element of self-concept development as individuals’ social perceptions tend to aggregate and stabilize through repeated interactions, providing them with a reliable framework for self-understanding (McCall and Simmons, 1978).

Furthermore, the stability of reflected appraisal may explain why it exhibits a weaker association with fluctuations in mood compared to self-appraisal. This suggests that external perspectives play a crucial role in offering a stable foundation for self-evaluation that the more dynamic nature of self-appraisal alone cannot provide. Previous studies support this observation, highlighting the variability of self-appraisal in everyday life and its connection to emotional states (Oosterwegel et al., 2001; Geukes et al., 2017). Earlier research underscores the influence of mood on cognition, suggesting that individuals may rely on their current emotional state when assessing their self-worth, as outlined by the feelings-as-information theory (Schwarz, 2011). According to this theory, mood can act as a reference point in making judgments about otherwise unrelated aspects, such as self-esteem. Consequently, the stability of reflected appraisal offers a consistent external perspective, making it less affected by mood fluctuations compared to the more dynamic self-appraisal. This stability helps to explain why reflected appraisal shows less variability with mood changes, aligning with previous findings that self-appraisal is more variable and closely tied to emotional states.

### 4.1 Limitations

Several limitations must be considered when interpreting the results of this study. The observed within variability in self-appraisal and reflected appraisal across the assessment period was smaller than what is expected of psychological constructs in EMA studies (Bolger and Laurenceau, 2013). On the one hand, it is crucial to recognize that a certain degree of stability in self-appraisal and reflected appraisal might be normative, particularly in healthy individuals, as consistent self-appraisal and perception of others’ appraisal may reflect a well-adjusted sense of self. Indeed, research suggests that healthy adults typically aim for stable self-concepts (Festinger et al., 1954; Swann, 2012) and show less variability compared to individuals with mental disorders such as Borderline-personality disorder (e.g., Santangelo et al., 2017a; Winter et al., 2017). On the other hand, the low variability observed could also signal that the items used might not be sufficiently sensitive to the nuanced fluctuations in self-appraisal and reflected appraisal that may occur. With standard deviations ranging from 1.6 to 1.9, the variability, while moderately present, may not be high enough to detect more subtle shifts in self-perception and external feedback. One potential reason for this could be the polarizing wording of the items, which may encourage more extreme responses and limit participants’ ability to express the full range of their self-evaluations. As a result, the items may fail to capture the dynamic and fluctuating nature of self-appraisal and reflected appraisal, leading to underrepresentation of important but subtle changes in self-appraisal and others’ appraisal. Future studies should aim to validate appropriate measures that can capture the variability of self-appraisal and reflected appraisal accurately, ensuring that these measures neither under-represent variability nor overestimate it. Additionally, *post hoc* analyses revealed that the variability of self-appraisal and reflected appraisal significantly decreased over the course of the assessment period. This could be due to participants becoming more familiar with the assessment process and adapting to the study context, leading to more stable responses over time (Bolger and Laurenceau, 2013). However, these decreases may also reflect a refinement in self-understanding, where participants develop clearer and more stable self-perceptions through repeated assessments, consistent with findings that EMA enhances self-awareness (Runyan et al., 2013). Over time, as participants mature in their understanding of self-evaluations and others’ perceptions, their responses may become more stable. This process may relate to Self-Concept Clarity (SCC), which involves stability, perceived internal consistency, and confidence in one’s self-concept (Campbell et al., 1996). While SCC is often associated with stable self-views (Campbell et al., 1996), repeated self-evaluation and external feedback may also facilitate its development, as individuals refine and integrate their self-perceptions over time. These results underscore the importance of longitudinal studies to explore these developmental trajectories in self-appraisal and reflected appraisal, as repeated self-evaluation may foster confidence by encouraging participants to refine and stabilize their self-perceptions over time, thus enhancing their understanding of both self-appraisal and reflected appraisal. Lastly, although Ecological Momentary Assessment (EMA) is a state-of-the-art approach for measuring real-time fluctuations in daily life, it comes with certain limitations. Specifically, the number of items assessed per time point is limited to reduce participant burden, which may restrict the depth of data collected. Future research should aim to explore momentary reasons for affective instability, such as interpersonal events or social stressors, to provide a more nuanced understanding. These factors should be addressed in future studies to build on the current findings and further refine the understanding of appraisal processes.

## 5 Conclusion

This study provides the first empirical evidence that reflected appraisal shows moderate fluctuation over several days, reflecting a normative pattern for healthy individuals. Unlike self-appraisal, reflected appraisal demonstrates greater stability over time, with slower deterioration and recovery, indicating a more stable yet less responsive evaluation process. Moreover, the findings reveal that instability in self-appraisal is more closely linked to mood instability than is reflected appraisal, suggesting that self-perception is more affected by internal fluctuations compared to the more stable, detached perspective of reflected appraisal.

Future research should delve into the dynamics of self-appraisal and reflected appraisal, particularly their long-term and short-term components. Investigating how long-term mood disorders affect the stability of these appraisal could provide insights into their relationship with self-concept, especially how discrepancies between self-appraisal and reflected appraisal impact mood in individuals with mental health challenges. This could inform therapeutic strategies aimed at enhancing emotional stability and self-concept coherence.

Integrating self- and reflected appraisal offers a comprehensive view of self-perception, illustrating how different perspectives of self-evaluation interact. This approach enhances our understanding of self-concept stability and the role of emotional states in shaping self-evaluations. Future studies should continue exploring these dynamics and their implications for mental health.

## Data Availability

The raw data supporting the conclusions of this article will be made available by the authors, without undue reservation.
