# Supplementary material for: Mirroring minds: assessing the relative stability of self-appraisal and reflected appraisal in daily life
Source: Front Psychol. 2025 Apr 25;16:1576353. doi: 10.3389/fpsyg.2025.1576353 (PMC12062088; doi:10.3389/fpsyg.2025.1576353)
Supplement: Supplementary file 1 [file Data_Sheet_1.docx]

Supplementary Material

# Supplementary Material^[[1]](#footnote-1)^

## Used Scales and their English translation

**Situativer Gedanke** (Bryant et al., 2013)

*Ich habe gerade über mich nachgedacht*

Ja/nein

*Ich habe gerade über eine/andere Person(en) nachgedacht*

Ja/nein

**Spontanous thought** (Bryant et al., 2013)

“I was just thinking about myself.”

yes/no

“I was just thinking about (an)other person(s).”

yes/no

**Stimmung** (Wilhelm & Schoebi, 2007)

Im Moment fühle ich mich …

sehr unruhig [0 - 1 - 2 - 3 - 4 - 5 - 6] sehr ruhig

sehr angespannt [0 - 1 - 2 - 3 - 4 - 5 - 6] sehr entspannt

sehr müde [0 - 1 - 2 - 3 - 4 - 5 - 6] sehr wach

sehr energielos [0 - 1 - 2 - 3 - 4 - 5 - 6] sehr energiegeladen

**Mood** (Wilhelm & Schoebi, 2007)

At this moment I feel …

very agitated [0 - 1 - 2 - 3 - 4 - 5 - 6] very calm

very tensed [0 - 1 - 2 - 3 - 4 - 5 - 6] very relaxed

very tired [0 - 1 - 2 - 3 - 4 - 5 - 6] very awake

without energy [0 - 1 - 2 - 3 - 4 - 5 - 6] full of energy

**Positiver and negativer Affekt** (Simons et al., 2020)

Im Moment fühle ich mich …

fröhlich [0 - 1 - 2 - 3 - 4 - 5 - 6]

zufrieden [0 - 1 - 2 - 3 - 4 - 5 - 6]

glücklich [0 - 1 - 2 - 3 - 4 - 5 - 6]

unsicher [0 - 1 - 2 - 3 - 4 - 5 - 6]

ängstlich [0 - 1 - 2 - 3 - 4 - 5 - 6]

niedergeschlagen [0 - 1 - 2 - 3 - 4 - 5 - 6]

schuldig [0 - 1 - 2 - 3 - 4 - 5 - 6]

**Positive and negative affect** (Simons et al., 2020)

At this moment I feel …

cheerful [0 - 1 - 2 - 3 - 4 - 5 - 6]

content [0 - 1 - 2 - 3 - 4 - 5 - 6]

happy [0 - 1 - 2 - 3 - 4 - 5 - 6]

insecure [0 - 1 - 2 - 3 - 4 - 5 - 6]

afraid [0 - 1 - 2 - 3 - 4 - 5 - 6]

down [0 - 1 - 2 - 3 - 4 - 5 - 6]

guilty [0 - 1 - 2 - 3 - 4 - 5 - 6]

**Selbsteinschätzung** (Collani & Herzberg, 2003; Santangelo, Koenig, et al., 2017)

Wie sehen Sie sich selbst in diesem Moment?

Trifft gar nicht zu – Trifft vollkommen zu              [0 - 1 - 2 - 3 - 4 - 5 - 6 - 7 - 8 - 9]

·         Ich bin mit mir selbst zufrieden.

·         Ich denke, dass ich gar nichts tauge.

·         Ich halte mich für einen Versager.

·         Ich halte mich für einen wertvollen Menschen.

**Self-appraisal** (Collani & Herzberg, 2003; Santangelo, Koenig, et al., 2017)

At the moment

strongly agree – strongly disagree              [0 - 1 - 2 - 3 - 4 - 5 - 6 - 7 - 8 - 9]

·         I am satisfied with myself.

·         I think I am no good at all.

·         I am inclined to feel that I am a failure.

·         I feel that I am a person of worth.

**Perspektivwechsel** (Collani & Herzberg, 2003; Santangelo, Koenig, et al., 2017)

Wie würden andere Menschen Sie in diesem Moment bewerten?

Trifft gar nicht zu – Trifft vollkommen zu              [0 - 1 - 2 - 3 - 4 - 5 - 6 - 7 - 8 - 9]

·         Andere Menschen sind zufrieden mit mir.

·         Andere Menschen denken, dass ich gar nichts tauge.

·         Andere Menschen halten mich für einen Versager.

·         Andere halten mich für einen wertvollen Menschen.

**Reflected Appraisal** (Collani & Herzberg, 2003; Santangelo, Koenig, et al., 2017)

How do others see you at this moment?

At this moment

strongly agree – strongly disagree              [0 - 1 - 2 - 3 - 4 - 5 - 6 - 7 - 8 - 9]

·         I am satisfied with myself.

·         I think I am no good at all.

·         I am inclined to feel that I am a failure.

·         I feel that I am a person of worth.

**Situativer sozialer Kontext**

Ich bin grade…

… alleine.

… mit anderen Menschen in Kontakt.

**Social Context**

At this moment I am …

… alone.

… in contact with others.

1. Scales not analyzed in the present article are included for documentation and transparency. [↑](#footnote-ref-1)
